# Educational and health outcomes of schoolchildren in local authority care in Scotland: A retrospective record linkage study

**DOI:** 10.1371/journal.pmed.1003832

**Published:** 2021-11-12

**Authors:** Michael Fleming, James S. McLay, David Clark, Albert King, Daniel F. Mackay, Helen Minnis, Jill P. Pell

**Affiliations:** 1 Institute of Health and Wellbeing, University of Glasgow, Glasgow, United Kingdom; 2 Department of Child Health, University of Aberdeen, Aberdeen, United Kingdom; 3 Public Health Scotland, Edinburgh, United Kingdom; 4 ScotXed, Scottish Government, Edinburgh, United Kingdom; Stellenbosch University, SOUTH AFRICA

## Abstract

**Background:**

Looked after children are defined as children who are in the care of their local authority. Previous studies have reported that looked after children have poorer mental and physical health, increased behavioural problems, and increased self-harm and mortality compared to peers. They also experience poorer educational outcomes, yet population-wide research into the latter is lacking, particularly in the United Kingdom. Education and health share a bidirectional relationship; therefore, it is important to dually investigate both outcomes. Our study aimed to compare educational and health outcomes for looked after children with peers, adjusting for sociodemographic, maternity, and comorbidity confounders.

**Methods and findings:**

Linkage of 9 Scotland-wide databases, covering dispensed prescriptions, hospital admissions, maternity records, death certificates, annual pupil census, examinations, school absences/exclusions, unemployment, and looked after children provided retrospective data on 715,111 children attending Scottish schools between 2009 and 2012 (13,898 [1.9%] looked after). Compared to peers, 13,898 (1.9%) looked after children were more likely to be absent (adjusted incidence rate ratio [AIRR] 1.27, 95% confidence interval [CI] 1.24 to 1.30) and excluded (AIRR 4.09, 95% CI 3.86 to 4.33) from school, have special educational need (SEN; adjusted odds ratio [AOR] 3.48, 95% CI 3.35 to 3.62) and neurodevelopmental multimorbidity (AOR 2.45, 95% CI 2.34 to 2.57), achieve the lowest level of academic attainment (AOR 5.92, 95% CI 5.17 to 6.78), and be unemployed after leaving school (AOR 2.12, 95% CI 1.96 to 2.29). They were more likely to require treatment for epilepsy (AOR 1.50, 95% CI 1.27 to 1.78), attention deficit hyperactivity disorder (ADHD; AOR 3.01, 95% CI 2.76 to 3.27), and depression (AOR 1.90, 95% CI 1.62 to 2.22), be hospitalised overall (adjusted hazard ratio [AHR] 1.23, 95% CI 1.19 to 1.28) for injury (AHR 1.80, 95% CI 1.69 to 1.91) and self-harm (AHR 5.19, 95% CI 4.66 to 5.78), and die prematurely (AHR 3.21, 95% CI 2.16 to 4.77). Compared to children looked after at home, children looked after away from home had less absenteeism (AIRR 0.35, 95% CI 0.33 to 0.36), less exclusion (AIRR 0.63, 95% CI 0.56 to 0.71), less unemployment (AOR 0.53, 95% CI 0.46 to 0.62), and better attainment (AIRR 0.31, 95% CI 0.23 to 0.40). Therefore, among those in care, being cared for away from home appeared to be a protective factor resulting in better educational outcomes. The main limitations of this study were lack of data on local authority care preschool or before 2009, total time spent in care, and age of first contact with social care.

**Conclusions:**

Looked after children had poorer health and educational outcomes than peers independent of increased neurodevelopmental conditions and SEN. Further work is required to understand whether poorer outcomes relate to reasons for entering care, including maltreatment and adverse childhood events, neurodevelopmental vulnerabilities, or characteristics of the care system.

## Abstract 2

In this record-linkage study in Scotland, Michael Fleming and colleagues compare educational and health outcomes for children who are in the care of their local authority with the outcomes of their peers.

## Author summary

**Why was this study done?:**

- Looked after children, defined as children placed in the care of their local authority, comprise a vulnerable cohort exhibiting poorer educational and health outcomes compared to peers.
- Only a few small sample studies have been conducted in the UK, and a recent systematic review reported an urgent need for more population-wide research into the educational outcomes of children placed in social care services.
- To our knowledge, no previous UK studies have dually investigated educational and health outcomes of looked after children compared to peers using an unselected, nationwide cohort.

**What did the researchers do and find?:**

- We linked together 9 Scotland-wide databases to compare educational and health outcomes of looked after children against peers, adjusting for sociodemographic, maternity, and comorbidity confounders.
- Looked after children had increased risk of school absenteeism and exclusion; special educational need (SEN); poor academic attainment; unemployment; hospitalisation and mortality; and treatment for epilepsy, attention deficit hyperactivity disorder (ADHD), depression, and neurodevelopmental multimorbidity.
- Among children in care, those looked after away from home had less absenteeism, less exclusion, less unemployment, and better attainment.

**What do these findings mean?:**

- Looked after children had poorer health and educational outcomes than peers independent of increased neurodevelopmental conditions and SEN.
- Among those in care, being cared for away from home appeared to be a protective factor resulting in better educational outcomes.
- Further work is required to understand whether poorer outcomes relate to reasons for entering care, including maltreatment and adverse childhood events, neurodevelopmental vulnerabilities, or characteristics of the care system.

## Introduction

Looked after children are defined as children who are placed in the care of their local authority [1]. Reasons for intervention include neglect, abuse or risk of abuse, complex disabilities/needs of children or parents, and involvement in crime [2]. Looked after children may live with parents under supervision of social workers, with family members, friends, foster carers, or prospective adopters or in residential schools/units or secure care [3,4]. In 2018, around 15,000 (1.4%) and 75,000 (0.6%) children were looked after in Scotland [4] and England [5], respectively. Estimating prevalence of looked after children across countries is complicated by limited data and differing ascertainment [6]. Previous studies report poorer mental and physical health [7–12], increased behavioural, psychosocial, and psychiatric problems, increased smoking, substance abuse, criminality, and teenage pregnancy [7,8,12–14], poorer dental health [15], and greater self-harm, suicidal behaviour [7,8,12–14], and mortality among looked after children [16]. While epilepsy, attention deficit hyperactivity disorder (ADHD), and depression commonly occur [7,10,11], atopic conditions like skin disorders and asthma are less prevalent [8,9].

Looked after children exhibit poorer educational outcomes including lower attainment, increased absenteeism, and exclusion [2,17,18]; however, Scottish analyses were not adjusted for confounders including poorer health [2]. A recent systematic review recommended more population-wide research into educational outcomes of looked after children [17]. Most studies comparing educational or health outcomes with peers have used small samples with larger ones focusing on absenteeism, academic attainment, or mortality [18–22]. While previous studies have compared both educational and health outcomes of looked after children with peers, to our knowledge, no population-wide studies have occurred in the UK despite Scotland’s ability to undertake population-wide linkage of administrative health, social care, and education records [3]. We aimed to address this gap.

Outcomes based on previous literature and available data included absenteeism, exclusion, special educational need (SEN), attainment, unemployment, all-cause/self-harm/injury admissions, prescribed medications, neurodevelopmental multimorbidity, and mortality. The bidirectional education–health relationship underpinned our rationale for investigating both sets of outcomes. We aimed to compare educational and health outcomes for looked after children with peers, adjusting for sociodemographic, maternity, and comorbidity confounders and hypothesised poorer outcomes among looked after children across all domains.

## Methods

This study is reported as per the Strengthening the Reporting of Observational Studies in Epidemiology (STROBE) guideline (S1 STROBE Checklist). While we did not publish an analysis plan, our analyses were planned in advance of the research team accessing any data. We subsequently added some additional analyses of type of placement within the looked after group at the request of the reviewers.

### Databases

We linked Scotland-wide, individual-level data from 4 health and 5 education databases, held by Public Health Scotland, and the Scottish Exchange of Educational Data (ScotXed), respectively. The pupil census, conducted annually by all local authority run primary, secondary, and special schools across Scotland, records demographic data including whether a child has a SEN and the type of SEN. The Children Looked After Survey (CLAS) records information on looked after children in the previous school year. Absences and exclusions, collected prospectively, are appended at the end of the school year. The Scottish Qualifications Authority collects examination attainment data for all Scottish schoolchildren. The school leaver database records pupils’ destination 6 months postschool: paid/voluntary employment, higher/further education, training, or unemployment. The Prescribing Information System (PIS) records prescriptions dispensed to Scottish residents by community pharmacies or primary care. Medications are classified using the British National Formulary (BNF) guidelines. The Scottish Morbidity Record (SMR) 02 maternity database collects data on maternal, obstetric, and child factors. SMR 01 and SMR 04 record acute and psychiatric admissions, including dates of admission and discharge and International Classification of Diseases-10th revision (ICD-10) diagnostic codes. National Records of Scotland collects death certificates, including date and cause of death. Scottish education and health records contain pupil-unique Scottish Candidate Numbers (SCNs) and patient-unique Community Health Index (CHI) numbers, respectively. Education records were linked together using SCN. Pupil census education records were probability matched against the CHI database (population-wide register of all patients in NHS Scotland) using sex, date of birth, and postcode. Linked and appended CHI numbers then enabled further deterministic (exact) matching of education records to maternity, prescribing, hospital admission, and mortality data described previously [23–31] (S1 Fig).

### Inclusion criteria, definitions, and outcomes

Our cohort comprised children attending Scottish schools between 2009 and 2012. We defined looked after children as looked after at anytime between 2009 and 2012. Our study was restricted to singletons born in Scotland and pupils aged ≥4 years and ≤18 years. Our study was restricted to singletons due to linkage limitations whereby we could not be sure that the correct child had linked to maternity data for multiple births of the same sex.

Annual school outcomes were number of days absent, number of exclusions for challenging/disruptive behaviour, and record of SEN. Absence and exclusion data were only available for 2009, 2010, and 2012. SEN is defined as unable to benefit from school education without help beyond that normally given to schoolchildren of similar age. The school census records any requirement for SEN for all children including type of SEN. These SEN records are a reliable source of data on certain neurodevelopmental disorders including autism and learning disability. We investigated all SEN attributed to physical and mental impairment/disability (S1 Table). Children could have more than 1 type recorded. Final school leaver outcomes were academic achievement and unemployment 6 months postschool, and these were available for a subset of children old enough to sit exams and leave school during the study period. Academic achievement was derived using number of awards attained at each level of the Scottish Credit Qualifications Framework (SCQF) and converted into an ordinal variable: low, basic, broad/general, and high attainment. Destination 6 months postschool was collapsed into a dichotomous variable: education/employment/training versus unemployment.

Health outcomes available for all children were hospital admission for any cause, injury, and self-harm; all-cause mortality; prescriptions for diabetes (insulin), asthma (inhaled corticosteroid and beta agonist both dispensed twice or more over 1 year), epilepsy (BNF section 4.8), ADHD (methylphenidate hydrochloride, dexamphetamine sulphate, atomoxetine, or lisdexamfetamine dimesylate), depression (tricyclic antidepressant, selective serotonin reuptake inhibitor, mirtazapine, or venlafaxine) and skin disorders (BNF sections 13.2.1, 13.4, and 13.5); and neurodevelopmental multimorbidity (defined as coexistence of 2 or more of epilepsy, ADHD, depression, autism, and learning disability where the first 3 were ascertained from prescribing records and the latter 2 were ascertained from records of SEN obtained from the school census). At the point when our data were linked, hospital admissions and deaths were available until September 2014 providing mean follow-up of 4.0 years (maximum 5.0 years).

We adjusted for confounders. The pupil census provided children’s age, sex, and ethnicity. Retrospective linkage to SMR02 provided maternal age at delivery, parity, maternal smoking, gestation at delivery, mode of delivery, 5-minute Apgar score, and child postcode at birth for those children born in Scotland. We derived sex-, gestation-specific birth weight centiles to measure intrauterine growth. Area socioeconomic deprivation was derived from postcode of residence at time of birth using the Scottish Index of Multiple Deprivation (SIMD) 2012 and then categorised into quintiles. SIMD is derived from 38 indicators across 7 domains (income, employment, health, housing, geographic access, crime and education, skills, and training) using information collected for data zones of residence (median population 769). We further adjusted main outcomes for presence of chronic conditions and SEN and then reran the models excluding these children.

### Approvals

The NHS West of Scotland Research Ethics Service confirmed that formal NHS ethics approval was not required since the study involved linkage of routinely collected data with an acceptably negligible risk of identification. The study was approved by the Public Benefit and Privacy Panel of NHS Public Health Scotland. A data processing agreement was drafted between Glasgow University and Public Health Scotland and a data sharing agreement between Glasgow University and ScotXed. The linked data were anonymised and then stored and analysed within the national safe haven.

### Public and patient involvement

It was not appropriate or possible to involve patients or the public in the design, conduct, reporting, or dissemination of our research.

### Dissemination declaration

We do not plan to disseminate the results directly to study participants and/or patient organisations.

### Statistical analyses

Characteristics of looked after children were compared with peers using chi-squared tests and chi-squared tests for trend. SEN, absences, and exclusions were analysed as yearly outcomes using population-averaged generalised estimating equations (GEEs) [32] adjusting for correlations between observations relating to the same pupil across different census years. The exposure (looked after in the previous school year) predated the absence, exclusion, and SEN outcomes (recorded for current school year). The user written quasi-likelihood under the independence model criterion (QIC) statistic compared different correlation structures. The structure with the lowest trace QIC was selected as most appropriate [33] Number of days absent and number of exclusions were modelled using longitudinal GEE analyses with a negative binomial distribution and log link function. Number of possible annual attendances was used as an offset variable to adjust for individual exposure time. SEN was modelled using GEE analyses with a binomial distribution and logit link.

Binary logistic regression modelled medication for diabetes, asthma, epilepsy, ADHD, depression or a skin disorder, and unemployment 6 months postschool. Ordinal logistic regression modelled final attainment and neurodevelopmental multimorbidity. The exposure (ever looked after at school during the study period) predated final attainment and subsequent unemployment outcomes. We could not, however, be certain about the specific temporal relationships between entering care (exposure) and initiation of prescribed medications (treatment). We explored whether absenteeism and exclusion mediated the association between looked after status and academic attainment by including them as covariates. We then performed formal mediation analyses using Stata’s *paramed* command [34]. We used the same approach to explore whether attainment mediated the association between looked after status and unemployment.

Subsequent hospitalisations (all-cause, injury, and self-harm) and all-cause mortality were modelled using Cox proportional hazard models. Looked after children were followed from the school census year where they were first recorded as being in care. Peers were followed from the date of their first school census year; therefore, the exposure predated all of the subsequent hospitalisation and mortality outcomes. Proportionality was tested using the Stata *estat phtest* command. We explored age, sex, and deprivation as effect modifiers, tested for statistical interactions, and undertook subgroup analyses where these were significant. For SEN, attainment, unemployment, hospitalisation, and mortality, we calculated population attributable fractions using the Stata *punaf* command to determine the extent they were explained by looked after status. Finally, within the subgroup of looked after children, we investigated the association between type of placement (at home versus away from home) and the 5 main educational outcomes. All *p*-values were <0.001 unless otherwise stated. All statistical analyses were undertaken using Stata MP version 14.1

## Results

Our linkage of education records to the CHI database achieved a linkage rate of 99.1%. Further linkage of these records to the SMR02 maternity database, achieved a subsequent linkage rate of 86.3%. After making further exclusions, 715,111 remaining singleton children born in Scotland attended a Scottish school for at least 1 year between 2009 and 2012, providing 2,237,671 pupil records (S2 Fig). Some pupils contributed only 1 year of schooling (if, for example, they were part of the 2009 census but left school before the 2010 census or if they only started school in 2012), while others were present across all years. Children could have been in any year of school and of any age (4 through 18 years) at the 2009 study start date. The mean number of observed school years per pupil was 3.1 (range 1.0 to 4.0), and the mean age of children across all observed years was 10.94 years [SD = 3.95]. In total, 13,898 (1.9%) children were looked after during the study period. Compared to peers, looked after children were younger (mean age across all school years 10.84 [SD = 3.40] versus 10.94 [SD = 3.96]), more likely to be male, more likely to be of white and less likely to be of Asian ethnicity, and more likely to live in more deprived areas, be treated for chronic conditions, have neurodevelopmental multimorbidity, and require all types of SEN. They had lower gestational age at delivery, lower birth weight centiles, and lower 5-minute Apgar scores. Their mothers were younger and more likely to have smoked during pregnancy and be multiparous (Table 1). The percentage of data missing within each variable was <0.25%, excluding parity (0.5%), Apgar score (1.0%), ethnicity (1.6%), and smoking during pregnancy (11.3%). Missing values for the latter 2, however, were analysed as “unknown” and included in all analyses.

**Table 1:** Characteristics of looked after schoolchildren compared with their peers.

	Not looked after		Looked after	
	*N* = 701,203		*N* = 13,898	
	*N*	%	*N*	%
**Sociodemographic factors**				
Sex				
Male	356,674	50.9	7,444	53.6
Female	344,539	49.1	6,454	46.4
Missing	0		0	
Average age across all school years				
Mean (SD)	10.94	3.96	10.84	3.40
Deprivation quintile				
1 (most deprived)	184,442	26.4	8,046	58.0
2	148,118	21.2	3,192	23.0
3	129,462	18.5	1,510	10.9
4	122,089	17.5	743	5.4
5 (least deprived)	115,431	16.5	374	2.7
Missing	1,671		33	
Ethnic group				
White	664,974	96.4	13,364	98.3
Asian	15,809	2.3	55	0.4
Black	1,430	0.2	17	0.1
Mixed	5,986	0.9	122	0.9
Other	1,840	0.3	31	0.2
Missing	11,174		309	
**Medication for comorbid conditions**				
Diabetes	3,001	0.4	41	0.3
Asthma	40,192	5.7	875	6.3
Epilepsy	4,419	0.6	147	1.1
ADHD	6,673	1.0	704	5.1
Depression	5,152	0.7	176	1.3
Skin disorder	108,103	15.4	1,875	13.5
**Total number of neurodevelopmental conditions**				
0	664,056	94.7	11,620	83.6
1	31,907	4.6	1,965	14.1
> = 2	5,250	0.8	313	2.2
**SEN at school**				
Autism	8,761	1.2	225	1.6
Learning disability	17,991	2.6	1,392	10.0
Learning difficulty	42,942	6.1	1,993	14.3
Sensory impairment	4,878	0.7	225	1.6
Physical motor disability	6,709	1.0	248	1.8
Communication problems	12,389	1.8	596	4.3
Social, emotional, or behavioural difficulty	23,994	3.4	3,879	27.9
Physical health condition	7,037	1.0	266	1.9
Mental health condition	1,418	0.2	133	1.0
Any of the above reasons	89,729	12.8	6,083	43.8
**Maternity factors**				
Maternal age (years)				
≤24	188,057	26.8	7,739	55.7
25 to 29	207,596	29.6	3,219	23.2
30 to 34	201,622	28.8	1,977	14.2
≥35	103,926	14.8	963	6.9
Missing	12		0	
Maternal smoking				
No	453,146	72.8	3,545	28.6
Yes	168,943	27.2	8,866	71.4
Missing	79,124		1,487	
Parity				
0	316,968	45.4	4,886	35.5
1	242,786	34.8	4,012	29.1
>1	137,800	19.8	4,878	35.4
Missing	3,659		122	
Mode of delivery				
SVD	473,736	67.6	10,600	76.3
Assisted vaginal	84,088	12.0	1,076	7.7
Breech vaginal	2,060	0.3	70	0.5
Elective CS	52,491	7.5	748	5.4
Emergency CS	88,691	12.6	1,402	10.1
Missing	147		2	
Gestation (weeks)				
< = 27	1,036	0.1	42	0.3
28 to 32	6,337	0.9	253	1.8
33 to 36	31,914	4.6	1,155	8.3
37	34,148	4.9	1,025	7.4
38	87,841	12.5	2,081	15.0
39	144,320	20.6	2,885	20.8
40	211,860	30.2	3,716	26.8
41	157,369	22.5	2,338	16.8
42	25,187	3.6	381	2.7
> = 43	690	0.1	11	0.1
Missing	511		11	
Sex gestation-specific birth weight centile				
1 to 3	28,324	4.0	1,274	9.2
4 to 10	62,338	8.9	2,107	15.2
11 to 20	83,287	11.9	2,202	15.9
21 to 80	412,768	58.9	7,101	51.2
81 to 90	60,121	8.6	655	4.7
91 to 97	37,913	5.4	399	2.9
98 to 100	15,607	2.2	137	1.0
Missing	855		23	
5-minute Apgar				
1 to 3	3,377	0.5	121	0.9
4 to 6	6,674	1.0	161	1.2
7 to 10	683,999	98.6	13,415	97.9
Missing	7163		201	

* Neurodevelopmental conditions include epilepsy, ADHD, depression, autism, and learning disability.

* All *p*-values <0.001 with exception of diabetes (*p* = 0.017), asthma (*p* = 0.005), and age (*p* = 0.017) following chi-squared tests for association, chi-squared tests for trend, and a *t* test (mean age).

ADHD, attention deficit hyperactivity disorder; CS, cesarean section; *N*, number; SVD, spontaneous vaginal delivery.

### Special educational need

**Fig 1:**
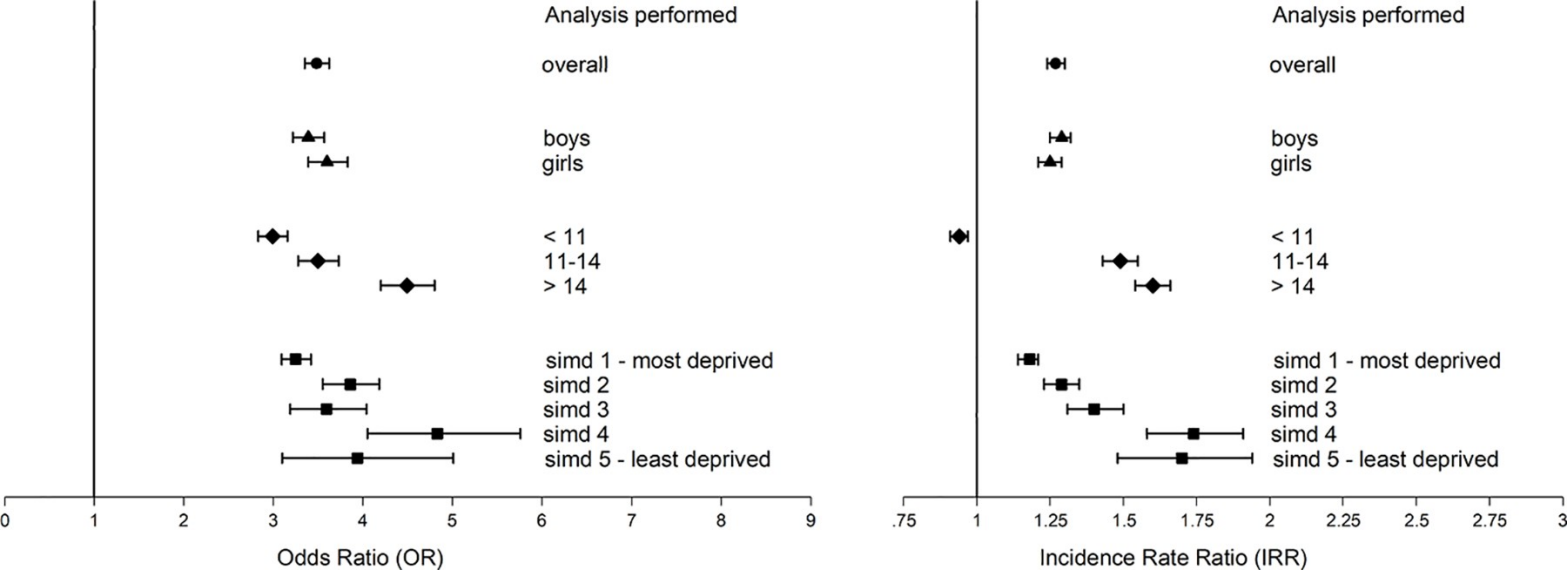
Forest plots of the association between being looked after at school and both SEN (left) and school absence (right) by sex, age, and deprivation. Adjusted for age, sex, deprivation quintile, ethnic group, maternal age, maternal smoking, parity, mode of delivery, gestation at delivery, sex gestation-specific birth weight centile, and 5-minute Apgar score. IRR, incidence rate ratio; OR, odds ratio; SEN, special educational need; SIMD, Scottish Index of Multiple Deprivation.

Compared with peers, looked after children had greater SEN after adjusting for sociodemographic and maternity factors (adjusted odds ratio [AOR] 3.48, 95% confidence interval [CI] 3.35 to 3.62) (Table 2). There were interactions with age and deprivation (both *p* < 0.001). Associations were stronger among older children, and the associations generally appeared stronger for more deprived children, although in this latter finding, the odds ratios were not monotonic, and the CIs were wide (Fig 1). Nevertheless, the latter could be attributable to greater baseline SEN in deprived areas among children not in care: 16.1% versus 9.1% in the most and least deprived quintiles, respectively. Among looked after children, SEN was also more common in the most (43.8%) versus least (38.5%) deprived quintile. Looked after children had greater SEN attributed to learning disability (AOR 2.70, 95% CI 2.51 to 2.91), learning difficulty (AOR 1.87, 95% CI 1.76 to 1.98), sensory impairment (AOR 1.65, 95% CI 1.40 to 1.95), physical motor disability (AOR 1.47, 95% CI 1.24 to 1.73), communication problems (AOR 2.06, 95% CI 1.85 to 2.30), social, emotional, and behavioural difficulties (AOR 6.46, 95% CI 6.15 to 6.78), physical health conditions (AOR 1.41, 95% CI 1.20 to 1.66), mental health conditions (AOR 3.52, 95% CI 2.78 to 4.44), and autism (AOR 1.22, 95% CI 1.04 to 1.44, *p* < 0.01).

**Table 2:** Association between being looked after at school and educational and health outcomes.

	Univariate model		Multivariable model 1^1^		Multivariable model 2^2^	
	Effect size	95% CI	Effect size	95% CI	Effect size	95% CI
	**IRR**		**IRR**		**IRR**	
Absence	1.82	1.78 to 1.86	1.49	1.46 to 1.52	1.27	1.24 to 1.30
Exclusion	9.08	8.60 to 9.59	6.18	5.84 to 6.53	4.09	3.86 to 4.33
	**OR**		**OR**		**OR**	
SEN	5.15	4.97 to 5.34	4.46	4.29 to 4.63	3.48	3.35 to 3.62
Attainment						
General/basic/low versus high	17.95	13.37 to 24.11	7.44	5.38 to 10.28	4.96	3.57 to 6.89
Basic/low versus general/high	10.51	8.94 to 12.37	5.51	4.49 to 6.75	4.00	3.26 to 4.91
Low versus basic/general/high	14.21	12.61 to 16.00	7.07	6.18 to 8.09	5.92	5.17 to 6.78
Unemployment	4.74	4.40 to 5.11	2.45	2.26 to 2.65	2.12	1.96 to 2.29
	**HR**		**HR**		**HR**	
All-cause admission	1.50	1.45 to 1.55	1.36	1.31 to 1.40	1.23	1.19 to 1.28
Injury admission	2.29	2.16 to 2.43	2.06	1.94 to 2.18	1.80	1.69 to 1.91
Self-harm admission	7.65	6.90 to 8.47	6.94	6.25 to 7.71	5.19	4.66 to 5.78
Mortality	4.16	2.86 to 6.07	3.68	2.51 to 5.40	3.21	2.16 to 4.77

^1^ Adjusted for sociodemographic (age, sex, deprivation quintile, and ethnic group) confounders.

^2^ Adjusted for sociodemographic (age, sex, deprivation quintile, and ethnic group) and maternity (maternal age, maternal smoking, parity, mode of delivery, gestation at delivery, sex gestation-specific birth weight centile, and 5-minute Apgar score) confounders.

All *p* < 0.001.

CI, confidence interval; HR, hazard ratio; IRR, incidence rate ratio; OR, odds ratio.

### School attendance

We analysed 1,597,397 available absence and exclusion records for 702,210 children with attendance data across 2009, 2010, and 2012. Looked after children had greater absenteeism compared to peers after adjusting for sociodemographic and maternity factors (adjusted incidence rate ratio [AIRR] 1.27, 95% CI 1.24 to 1.30) (Table 2). There were interactions with age and deprivation (both *p* < 0.001). The association was stronger among children aged >14 years (AIRR 1.60, 95% CI 1.54 to 1.66) than 11 to 14 years (AIRR 1.49, 95% CI 1.43 to 1.55) and <11 years (AIRR 0.94, 95% CI 0.91 to 0.97) and children in the least (AIRR 1.70, 95% CI 1.48 to 1.94) versus most (AIRR 1.18, 95% CI 1.14 to 1.21) deprived quintile (Fig 1). The latter was attributable to greater baseline yearly absenteeism among the most versus least deprived children not in care (median 11 versus median 5 days). Among looked after children, absences were also higher in the most versus least deprived quintiles (median 9.5 versus median 8 days).

**Fig 2:**
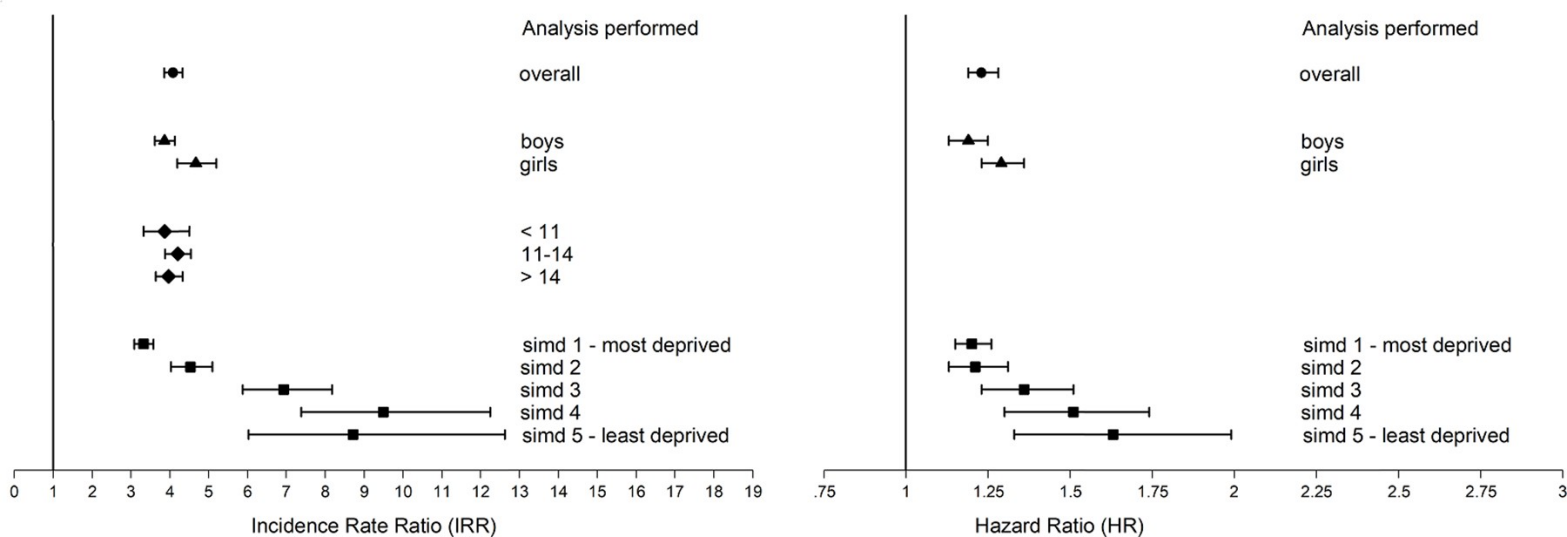
Forest plots of the association between being looked after at school and both school exclusion (left) and hospital admission (right) by sex, age, and deprivation. Adjusted for age, sex, deprivation quintile, ethnic group, maternal age, maternal smoking, parity, mode of delivery, gestation at delivery, sex gestation-specific birth weight centile, and 5-minute Apgar score. HR, hazard ratio; IRR, incidence rate ratio; SIMD, Scottish Index of Multiple Deprivation.

Exclusion from school was greater among looked after children after adjusting for sociodemographic and maternity confounders (AIRR 4.09, 95% CI 3.86 to 4.33) (Table 2). There were interactions with sex and deprivation (both *p* < 0.001). The association was stronger among girls (AIRR 4.67, 95% CI 4.19 to 5.20) than boys (AIRR 3.86, 95% CI 3.61 to 4.13) and among children in the least (AIRR 8.72, 95% CI 6.03 to 12.62) versus most (AIRR 3.32, 95% CI 3.08 to 3.58) deprived quintile (Fig 2). The latter was explained by greater baseline exclusion among the most versus least deprived children not in care (6.2% versus 1.1%). Among looked after children, exclusions were also more common in the most (22.1%) versus least (18.7%) deprived quintile.

### Academic attainment and school leaver unemployment

Of the 94,037 children in our cohort who were old enough to sit exams during the study period, looked after children were more likely to achieve the lowest level of academic attainment after adjustment for sociodemographic and maternity factors (AOR 5.92, 95% CI 5.17 to 6.78) (Table 2). There was an interaction with deprivation (*p* < 0.001). The association was stronger in the least (AOR 10.34, 95% CI 5.51 to 19.40) versus most (AOR 4.87, 95% CI 4.07 to 5.83) deprived quintile explained by poorer baseline attainment among more deprived children not in care: 8.27% of the most deprived quintile had the lowest level of attainment compared to 1.03% in the least deprived. This was similar among looked after children: 40.18% versus 32.56% in most and least deprived quintiles, respectively. Adjusting for absenteeism attenuated the main association (AOR 5.85, 95% CI 5.01 to 6.83). On formal mediation analysis, the association between looked after status and attainment (AOR 5.23 CI 4.09 to 7.31) was likely a direct effect (AOR 5.43 CI 4.28 to 7.47) rather than an indirect effect (AOR 0.96 CI 0.92 to 1.01, *p* = 0.292) mediated by absenteeism. Adjusting for exclusions also attenuated the main association (AOR 5.35, 95% CI 4.64 to 6.16). On formal mediation analysis, most of the association between looked after status and attainment (AOR 4.85 CI 3.70 to 6.59) comprised a direct effect (AOR 4.18 CI 3.19 to 5.80), with a smaller indirect effect mediated by exclusion (AOR 1.16 CI 1.13 to 1.21).

Of the 175,637 children in our cohort who left school during the study period, 2,487 (80.8%) of 3,079 looked after children left before 16 years of age, compared with 49,779 (28.9%) of 172,558 peers (*p* < 0.001). Looked after status was associated with increased unemployment after adjusting for sociodemographic and maternity factors (AOR 2.12, 95% CI 1.96 to 2.29) (Table 2). Adjusting for attainment attenuated the main association (AOR 1.45, 95% CI 1.26 to 1.66). On formal mediation analysis, the association between looked after status and unemployment (AOR 2.35 CI 2.05 to 2.79) comprised a direct effect (AOR 1.97 CI 1.74 to 2.33) and a smaller indirect effect mediated by attainment (AOR 1.19 CI 1.16 to 1.23).

### Hospitalisation and mortality

Linkage to hospital records provided 2.87 million person years of follow-up (mean follow-up time = 4.01 years). Of the 715,111 schoolchildren in our full cohort, 152,908 experienced 299,235 hospital admissions. The proportionality assumption was met for all Cox proportional hazards models. Looked after children had greater hospitalisation for any cause (adjusted hazard ratio [AHR] 1.23, 95% CI 1.19 to 1.28), injury (AHR 1.80, 95% CI 1.69 to 1.91), and self-harm (AHR 5.19, 95% CI 4.66 to 5.78) after adjustment for sociodemographic and maternity factors (Table 2). There were interactions with sex and deprivation whereby the risk of all-cause hospitalisation was marginally greater among girls (AHR 1.29, 95% CI 1.23 to 1.36) than boys (AHR 1.19, 95% CI 1.13 to 1.25) and greater among children in the least (AHR 1.63, 95% CI 1.33 to 1.99) versus most (AHR 1.20, 95% CI 1.15 to 1.26) deprived quintile (Fig 2). The latter finding was due to greater baseline hospitalisation in deprived areas among children not in care: 24.6% of the most deprived quintile were hospitalised for any cause compared with 18.0% of the least deprived. Among looked after children, hospitalisations were as common in the most (25.6%) and least (25.9%) deprived quintiles. The association with all-cause admission attenuated after further adjusting for unemployment, academic attainment, history of school exclusion, and school absence rate (AHR 1.16, 95% CI 1.04 to 1.30, *p* = 0.006). There were 488 deaths (47 suicides) over the study period: 29 (5 suicides) among looked after children and 459 (42 suicides) among peers. Looked after children had increased mortality over follow-up after adjusting for sociodemographic and maternity factors (AHR 3.21, 95% CI 2.16 to 4.77) (Table 2). The association attenuated after further adjusting for previous injury and self-harm admissions, unemployment, history of school exclusion, and school absence rate (AHR 2.70, 95% CI 1.36 to 5.37, *p* = 0.005).

### Health conditions

Looked after children were more likely to be treated for epilepsy (AOR 1.50, 95% CI 1.27 to 1.78), ADHD (AOR 3.01, 95% CI 2.76 to 3.27), and depression (AOR 1.90, 95% CI 1.62 to 2.22) and have neurodevelopmental multimorbidity (AOR 2.45, 95% CI 2.34 to 2.57) after adjusting for sociodemographic and maternity factors. Looked after status was not associated with treatment for diabetes (AOR 0.75, 95% CI 0.54 to 1.03, *p* = 0.078), asthma (AOR 0.94, 95% CI 0.87 to 1.01, *p* = 0.069), or a skin disorder (AOR 0.98, 95% CI 0.94 to 1.04, *p* = 0.529).

### Sensitivity analyses and population attributable fractions

Results from our main analyses remained significant, and effect sizes of similar magnitude, when we adjusted for, then excluded children treated for epilepsy, ADHD or depression, or who had SEN (S2 Table).

After adjusting for sociodemographic and maternity factors, population attributable fractions determined the extent to which looked after status explained each outcome (Table 3): 7.9% of self-harm admissions, 1.1% of injury admissions, 3.6% of SEN, 6.8% of children achieving the lowest level of academic attainment, 2.3% of school leaver unemployment, and 3.4% of deaths were attributed to children being in care.

**Table 3:** PAFs for looked after status.

	Unadjusted			Adjusted		
	PAF	95% CI	% Attributed	PAF	95% CI	% Attributed
SEN	0.045	(0.044 to 0.046)	4.5	0.036	(0.035 to 0.037)	3.6
Lowest level of academic attainment	0.091	(0.084 to 0.0.098)	9.1	0.068	(0.061 to 0.075)	6.8
Unemployment	0.040	(0.038 to 0.043)	4.0	0.023	(0.020 to 0.026)	2.3
All-cause hospital admission	0.003	(0.003 to 0.004)	0.3	0.000	(−0.001 to 0.001)	0.0
Injury admission	0.016	(0.014 to 0.018)	1.6	0.011	(0.009 to 0.013)	1.1
Self-harm admission	0.095	(0.085 to 0.106)	9.5	0.079	(0.069 to 0.089)	7.9
Mortality	0.041	(0.019 to 0.062)	4.1	0.034	(0.013 to 0.056)	3.4

Adjusted for age, sex, deprivation, ethnicity, maternal age, maternal smoking, model delivery, parity, gestation, birth weight, and 5-minute Apgar score.

CI, confidence interval; PAF, population attributable fraction; SEN, special educational need.

### Subgroup analyses of looked after children

Of 13,898 looked after children, 12,243 had placement type recorded, of whom 5,137 were always looked after at home, 6,712 away from home, and 394 experienced spells in both care settings (S3 Table). They spent a total of 27,030 years in care: 37.9% of these years were spent at home with parents, 26.4% with friends/relatives, 30.2% with foster carers, and 5.5% in other settings. Compared to children looked after at home, children looked after away from home had less absenteeism, exclusion, unemployment, and better attainment (S4 Table).

## Discussion

### Main findings

Compared with peers, looked after children had more school absences and exclusions, greater SEN, poorer examination grades, and increased unemployment postschool. Previous studies report similar patterns around absenteeism, exclusion, and attainment [17–18]. Our findings regarding unemployment and SEN are novel but not surprising given reports of poorer physical and mental health [7–12]. Looked after children in our cohort had increased SEN attributed to all types. Previous studies have reported increased behavioural issues, psychosocial and psychiatric problems, and risky behaviours [7,8,12–14]. Associations with absenteeism, exclusion, and SEN were stronger among less deprived children, explained by higher baseline prevalence of these outcomes among children not in care living in deprived areas.

We reported greater neurodevelopmental and mental health conditions among looked after children, yet no increased risk of physical conditions such as diabetes, asthma, or skin disorders. Previous studies have reported fewer skin disorders and atopic conditions among children in care [8,9]. Increased epilepsy, ADHD, and depression concurs with previous studies [7,10,11]. Neurodevelopmental conditions commonly overlap, share genetic aetiology, and are highly heritable [35], and we demonstrated higher risk of neurodevelopmental multimorbidity. We reported that looked after children were more likely to be hospitalised, for any cause, injury and self-harm, and die prematurely. Previous studies have observed increased self-harm, suicidal behaviour [7,12–14], and mortality [16].

We calculated population attributable fractions to crudely understand the contribution of looked after care to our observed outcomes. We attributed 7.9% of self-harm admissions and 6.8% of children achieving the lowest level of academic attainment to being in care. These percentages were higher than for other outcomes investigated. This is not immediately obvious why and should be investigated further. We previously reported that children treated for ADHD, depression, and epilepsy have poorer educational and health outcomes including injury and self-harm [24–27]. Higher observed prevalence of these conditions among looked after children is one plausible explanation for poorer outcomes observed in this study. However, our observed associations remained after adjusting for presence of these conditions. Alternative explanations include maltreatment and early life adversity [36,37] and components and characteristics of the care system itself [38].

On subgroup analyses of looked after children, we observed that, versus being looked after at home, children looked after away from home had less absenteeism, exclusion and unemployment, and better attainment. Being cared for away from home therefore appeared to be a protective factor resulting in better educational outcomes among those in care.

### Strengths

Ours was a large, nonselective study including children across the whole of Scotland. Missing data within each variable did not exceed 1.0% and, for most, were considerably lower. Given the low percentage of missing data and the large sample size, we do not believe this affected the results and so did not perform sensitivity analyses or data imputation. Because the sampling frame was all local authority maintained mainstream and special schools, inclusion was not restricted to children from specific backgrounds or geographical areas. The large study size provided power to test for statistical interactions and undertake subgroup analyses, and we analysed a range of outcomes spanning education, health, employment, and death. A recent systematic review not only recommended a need for more population-wide research into educational outcomes of looked after children but also described limitations regarding the relatively small number of existing UK peer-reviewed studies including small samples, poor or no adjustment for confounders, and an imbalanced focus on attainment to the detriment of other equally important educational and indeed health outcomes [17]. We addressed this gap by adjusting for potential confounders: sociodemographic, maternity, and comorbid conditions and investigated whether associations were independent of physical and mental health. Being in care can impact both health and educational outcomes, which, independently, share a bidirectional relationship. This interconnection was our rationale for investigating and adjusting for both and is a clear strength versus studies investigating one or the other.

### Limitations

We used existing, administrative databases established for other purposes. However, they undergo regular quality assurance checks. Linkage of education and health records relied on probabilistic matching; however, this has been validated as 99% accurate for singletons [23]. Our study did not cover private schools, but they account for less than 5% of schoolchildren. Additionally, 0.9% of children could not be linked to the CHI database, and a further 14.5% of those who did link to the CHI database could not be linked to Scottish maternity records. The percentage of children who were looked after was 1.9% among those who could not be linked to Scottish maternity records and was also 1.9% among those who could be linked suggesting bias was unlikely. Wider demographics were roughly similar among those who could versus could not be linked with respect to sex (50.9% boys versus 51.3% boys), age (mean 10.90 [SD = 3.97] versus 10.80 [SD = 4.09]), and deprivation (percentage in least deprived quintile 20.2% versus 21.6%; percentage in most deprived quintile 19.9% versus 18.9%); however, there were more children of white ethnicity among those who linked (94.7%) than did not (80.7%). Many of the nonlinkers were likely born outside Scotland. According to the 2011 Scottish Census, 11% of Scottish residents aged 5 to 19 years were born outside Scotland, consistent with 14.5% of Scottish children we could not link to Scottish maternity records.

A cohort approach ensured that initiation of looked after status predated the majority of the education and health outcomes. The exception to this is that we could not be certain about the specific temporal relationships between entering care and initiation of prescribed medications for the chronic conditions presented, meaning reverse causation is possible whereby conditions such as ADHD could contribute to difficulties that result in becoming looked after. Additionally, as reported previously, while ascertainment of children with type 1 diabetes based on receipt of necessary insulin was previously validated as 95% accurate [28], ascertaining children with asthma [29], epilepsy [26], ADHD [25], depression [27], and skin disorders [30] via prescriptions has limitations because some medications are used for alternative reasons and not all children are medicated. We had no access to primary care records and so could not confirm the underlying clinical indications for receipt of these medications. This issue is particularly apparent for antidepressants, which can be prescribed for other conditions including anxiety and obsessive-compulsive disorder. However, previous studies have reported depression to be the main reason for prescribing selective serotonin reuptake inhibitors [39–42] and 62.4% and 62.2% of children prescribed fluoxetine and citalopram to be depressed [40]. In our own previous study investigating educational and health outcomes of children prescribed antidepressants using similar methodology, our original associations remained after limiting ascertainment to children receiving solely fluoxetine or citalopram, reducing the chances of associations being affected by misclassification. Fluoxetine and citalopram are the recommended treatment and most common second-line treatment, respectively, in children under 16, and fluoxetine is the only drug licensed in the UK for treating depression in this age group.

We could not adjust for early life maltreatment and preschool social care factors, parental influence, and parental physical/mental health conditions, addiction problems, and/or criminality. Health compromises following maltreatment and early life trauma may not emerge for many decades; therefore, reporting short-term health outcomes in childhood and adolescence provides a restricted perspective resulting in likely underreporting of poor outcomes. This may have accounted for lack of association with some of the conditions investigated, and longer-term follow-up should be the focus of future work. While follow-up for interactions with the justice system was not the focus of our study, analysing these outcomes as well as longer-term employment outcomes would also add value in future work. A further limitation may arise through underreporting of neurodevelopmental disorders among looked after children because problems are attributed to early life abuse and neglect [43]. With respect to self-harm, our study could only ascertain self-harm episodes that resulted in hospitalisation, therefore excluding cases that were untreated or treated within accident and emergency or outpatient clinics.

We did not have any information on looked after status before 2009 or before starting school. Therefore, looked after children who came out of care before starting school and looked after children who came out of care before 2009 would not be ascertained as ever having been in care if they did not re-enter the system during our study period. Our exposed group may therefore have been reduced, and this would have resulted in us potentially underestimating the adverse effects of being in care on educational and health outcomes. However, our overall conclusions would remain unchanged. Access to more historic, and, in particular, preschool, social care data would help to solve this issue in future studies.

In our main analyses, looked after children comprised a heterogeneous group who had ever been looked after during the study period regardless of type of intervention, duration of care, or reasons for care. We conducted subgroup analyses to additionally investigate educational outcomes pertaining to children looked after at home versus not at home. However, future work should also consider factors that we were unable to investigate due to lack of reliable data, but which are likely to influence outcomes, namely total time spent in care and age of first contact with social care. The large sample size contributed to small *p*-values (<0.001). Some of the findings, therefore, while highly significant, represented fairly modest differences in real terms, which should be acknowledged during interpretation. Finally, generalisability to other studies may be limited for 3 main reasons. First, to other countries, where reasons for entry to care may differ. Second, to previous studies, which failed to adjust for important confounders adequately or at all. Third, due to heterogeneity in recording of educational and health outcomes between studies.

Despite these limitations, our findings highlight that those children who become looked after during their school years are at high educational risk compared to peers. It has been previously demonstrated that lower levels of educational achievement and fewer years of schooling are associated with poorer health and greater premature mortality [44,45], so it is essential that this vulnerable group of children receive additional educational focus. New initiatives such as “virtual schools” [46] have been designed to achieve this, and our findings reinforce the need for such additional measures if these children are to reach their full potential. However, the higher risk of neurodevelopmental multimorbidity, depression, self-harm, and premature mortality in this group suggests that closer involvement between child and adolescent mental health services and schools will also be crucial if this group is to receive appropriate mental health support and fully access the school curriculum. Our findings suggest that being placed in care away from home might ameliorate at least some poor outcomes in this group, but future research should focus on whether, and in what way, poorer outcomes are related to characteristics of the care system or precare factors, including neurodevelopmental vulnerabilities, maltreatment, adverse childhood events, and parental neurodevelopmental or psychiatric disorder. This will involve trying to access some of the data sources that we were unable to analyse during this study, such as preschool social care data, primary care data, and data around early life maltreatment and parental factors and influences. Longer-term follow-up, including linkage to wider sources such as justice and welfare data, would help uncover longer-term outcomes that we could not detect during this research. Access to richer data around duration of placement, age at first contact, and reasons for entry to care should be a priority to help uncover more around the specific drivers that influence poor outcomes.

## Conclusions

Compared to peers, looked after children were more likely to be absent and excluded from school, have SEN, perform poorly in exams, and be unemployed after leaving school. They were more likely to be treated for epilepsy, ADHD, and depression, have neurodevelopmental multimorbidity, be hospitalised for any cause, injury and self-harm, and die prematurely. Poorer outcomes were not explained by higher prevalence of diagnosed neurodevelopmental conditions and worse mental health. Our study unfortunately could not investigate characteristics of the care system or reasons for entering care, including maltreatment, adverse childhood events, and parental neurodevelopmental or psychiatric disorder. Therefore, further work is required to understand whether their poorer outcomes also relate to these factors and in what way. However, what is clear from our findings is that looked after children experience extreme educational disadvantage and therefore require specific educational focus if they are to fulfill their potential.

## Supporting information

S1 STROBE Checklist Statement—checklist of items that should be included in reports of observational studies. STROBE, Strengthening the Reporting of Observational Studies in Epidemiology.
(DOCX)

S1 Table SEN categories. SEN, special educational need.
(DOCX)

S2 Table Association between being looked after at school and educational and health outcomes after further adjustment for potential confounders. (DOCX)

S3 Table Type of placement and total number of years in care among 13,898 looked after children. (DOCX)

S4 Table Type of placement and educational outcomes among looked after children. (DOCX)

S1 Fig Linkage of education to health data. (TIF)

S2 Fig Linkage flow diagram detailing number of pupils included and excluded at each stage of data cleaning. (TIF)

## Abbreviations

ADHD: attention deficit hyperactivity disorder
AHR: adjusted hazard ratio
AIRR: adjusted incidence rate ratio
AOR: adjusted odds ratio
BNF: British National Formulary
CHI: Community Health Index
CI: confidence interval
CLAS: Children Looked After Survey
GEE: generalised estimating equation
ICD-10: International Classification of Diseases-10th revision
PIS: Prescribing Information System
QIC: quasi-likelihood under the independence model criterion
SCN: Scottish Candidate Number
SCQF: Scottish Credit Qualifications Framework
ScotXed: Scottish Exchange of Educational Data
SEN: special educational need
SIMD: Scottish Index of Multiple Deprivation
SMR: Scottish Morbidity Record
STROBE: Strengthening the Reporting of Observational Studies in Epidemiology
