# Supplementary material for: Educational and health outcomes of schoolchildren in local authority care in Scotland: A retrospective record linkage study
Source: PLoS Med. 2021 Nov 12;18(11):e1003832. doi: 10.1371/journal.pmed.1003832 (PMC8589203; doi:10.1371/journal.pmed.1003832)
Supplement: S1 Table — SEN, special educational need. (DOCX) [file pmed.1003832.s002.docx]

**S1 Table. Special educational need categories**

| **SEN code** | **SEN description** | **Derived SEN group** |
| --- | --- | --- |
| 10 | Learning disability | Learning disability |
| 11 | Dyslexia | learning difficulty |
| 12 | Other specific learning difficulty *(e.g. numeric)* |  |
| 13 | Other moderate learning difficulty |  |
| 20 | Visual impairment | sensory impairment |
| 21 | Hearing impairment |  |
| 22 | Deafblind |  |
| 23 | Physical or motor impairment | physical motor impairment |
| 24 | Language or speech disorder | communication problems |
| 44 | Communication support needs* |  |
| 25 | Autistic spectrum disorder | ASD |
| 26 | Social, emotional, and behavioural difficulty | Social, emotional, and behavioural difficulty |
| 27 | Physical health problem | Physical health problem |
| 28 | Mental health problem | Mental health problem |
| 40 | Interrupted learning | not classed as SEN |
| 41 | English as an additional language |  |
| 42 | Looked after |  |
| 43 | More able pupil |  |
| 45 | Young carer* |  |
| 46 | Bereavement* |  |
| 47 | Substance misuse* |  |
| 48 | Family issues* |  |
| 49 | Risk of exclusion* |  |
| 98 | Not disclosed/declared |  |
| 99 | Other |  |

* code only included in student need dataset from 2010 onwards
