# Supplementary material for: Educational and health outcomes of schoolchildren in local authority care in Scotland: A retrospective record linkage study
Source: PLoS Med. 2021 Nov 12;18(11):e1003832. doi: 10.1371/journal.pmed.1003832 (PMC8589203; doi:10.1371/journal.pmed.1003832)
Supplement: S2 Table — (DOCX) [file pmed.1003832.s003.docx]

**S2 Table. Association between being looked after at school and educational and health outcomes after further adjustment for potential confounders**

|  | **Multivariable Model 2 ^1^** | | **Multivariable Model 3 ^2^** | | **Multivariable Model 4 ^3^** | |
| --- | --- | --- | --- | --- | --- | --- |
|  | Effect | 95% CI | Effect | 95% CI | Effect | 95% CI |
|  | Size |  | Size |  | Size |  |
|  | **IRR** |  | **IRR** |  | **IRR** |  |
| Absence | 1.27 | 1.24-1.30 | 1.22 | 1.19-1.24 | 1.29 | 1.25-1.32 |
| Exclusion | 4.09 | 3.86-4.33 | 3.20 | 3.01-3.39 | 4.17 | 3.85-4.52 |
|  | **OR** |  | **OR** |  | **OR** |  |
| SEN | 3.48 | 3.35-3.62 | 3.35 | 3.22-3.50 | 3.47 | 3.33-3.62 |
| Attainment |  |  |  |  |  |  |
| general/basic/low versus high | 4.96 | 3.57-6.89 | 4.28 | 3.07-5.96 | 4.51 | 3.12-6.51 |
| basic/low versus general/high | 4.00 | 3.26-4.91 | 3.33 | 2.70-4.10 | 3.47 | 2.73-4.40 |
| low versus basic/general/high | 5.92 | 5.17-6.78 | 4.68 | 4.07-5.39 | 5.93 | 4.97-7.08 |
| Unemployment | 2.12 | 1.96-2.29 | 2.00 | 1.84-2.16 | 2.11 | 1.90-2.35 |
|  | **HR** |  | **HR** |  | **HR** |  |
| All-cause admission | 1.23 | 1.19-1.28 | 1.12 | 1.08-1.16 | 1.19 | 1.13-1.25 |
| Injury admission | 1.80 | 1.69-1.91 | 1.68 | 1.58-1.78 | 1.82 | 1.67-1.98 |
| Self-harm admission | 5.19 | 4.66-5.78 | 4.56 | 4.08-5.09 | 5.51 | 4.77-6.37 |
| Mortality | 3.21 | 2.16-4.77 | 2.25 | 1.51-3.35 | 4.22 | 2.32-7.67 |

^1^ adjusted for sociodemographic and maternity confounders*

^2^ adjusted for sociodemographic and maternity confounders and children treated for epilepsy, ADHD, depression or receipt of SEN

^3^ adjusted for sociodemographic and maternity confounders excluding children treated for epilepsy, ADHD, depression or in receipt of SEN

*Confounders include age, sex, deprivation quintile, ethnic group, maternal age, maternal smoking, parity, mode of delivery, gestation at delivery,

Sex-gestation-specific birthweight centile and 5-minute Apgar score

All p<0.001

IRR Incidence Rate Ratio; OR Odds Ratio; HR Hazard Ratio; CI confidence interval
