# Supplementary material for: Educational and health outcomes of schoolchildren in local authority care in Scotland: A retrospective record linkage study
Source: PLoS Med. 2021 Nov 12;18(11):e1003832. doi: 10.1371/journal.pmed.1003832 (PMC8589203; doi:10.1371/journal.pmed.1003832)
Supplement: S3 Table — (DOCX) [file pmed.1003832.s004.docx]

**S3 Table. Type of placement and total number of years in care among 13,898 looked-after-children**

|  | **Number** | **Percentage** |
| --- | --- | --- |
|  |  |  |
| At home with parents | 8,450 | 37.9 |
| With friends/relatives | 5,881 | 26.4 |
| Foster carers provided by local authority | 5,002 | 22.4 |
| Foster carers purchased by local authority | 1,750 | 7.8 |
| Permanent placement with prospective adopters | 128 | 0.6 |
| In other community | 21 | 0.1 |
| In local authority home | 614 | 2.8 |
| In voluntary home | 121 | 0.5 |
| In residential school | 158 | 0.7 |
| In secure accommodation | 19 | 0.1 |
| Crisis care | 21 | 0.1 |
| Other residential | 150 | 0.7 |
| Missing | 4,715 |  |
| **Total** | **27,030** | **100** |
|  |  |  |

Each child was looked after for a minimum of 1 year and a maximum of 4 years over the study period

The placement destination can vary year on year
