# Supplementary material for: Educational and health outcomes of schoolchildren in local authority care in Scotland: A retrospective record linkage study
Source: PLoS Med. 2021 Nov 12;18(11):e1003832. doi: 10.1371/journal.pmed.1003832 (PMC8589203; doi:10.1371/journal.pmed.1003832)
Supplement: S4 Table — (DOCX) [file pmed.1003832.s005.docx]

**S4 Table. Type of placement and educational outcomes among looked-after-children**

|  | type of placement (reference = at home) | | | |  |  |  |  |  |  |
| --- | --- | --- | --- | --- | --- | --- | --- | --- | --- | --- |
|  |  |  |  |  |  |  |  |  |  |  |
|  |  |  |  |  |  |  |  |  |  |  |
| Outcome | category | effect size | 95% CI | p value |  |  |  |  |  |  |
|  |  |  |  |  |  |  |  |  |  |  |
|  |  | **IRR** |  |  |  |  |  |  |  |  |
| Absence |  |  |  |  |  |  |  |  |  |  |
|  | away from home | 0.35 | 0.33-0.36 | <0.001 |  |  |  |  |  |  |
|  |  |  |  |  |  |  |  |  |  |  |
|  |  |  |  |  |  |  |  |  |  |  |
| Exclusion | |  |  |  |  |  |  |  |  |  |
|  | away from home | 0.63 | 0.56-0.71 | <0.001 |  |  |  |  |  |  |
|  |  |  |  |  |  |  |  |  |  |  |
|  |  |  |  |  |  |  |  |  |  |  |
|  |  | **OR** |  |  |  |  |  |  |  |  |
| SEN |  |  |  |  |  |  |  |  |  |  |
|  | away from home | 0.95 | 0.87-1.03 | NS |  |  |  |  |  |  |
|  |  |  |  |  |  |  |  |  |  |  |
|  |  |  |  |  |  |  |  |  |  |  |
| Attainment | |  |  |  |  |  |  |  |  |  |
|  | away from home | 0.31 | 0.23-0.40 | <0.001 |  |  |  |  |  |  |
|  | both* | 0.74 | 0.26-2.16 | NS |  |  |  |  |  |  |
|  |  |  |  |  |  |  |  |  |  |  |
| Unemployment | |  |  |  |  |  |  |  |  |  |
|  | away from home | 0.53 | 0.46-0.62 | <0.001 |  |  |  |  |  |  |
|  | both* | 0.73 | 0.45-1.19 | NS |  |  |  |  |  |  |
|  |  |  |  |  |  |  |  |  |  |  |
|  |  |  |  |  |  |  |  |  |  |  |
| Adjusted for age, sex, deprivation, ethnicity, maternal age, maternal smoking, model delivery, parity, gestation, birthweight and 5-minute Apgar score | | | | | | | | | | |
| IRR Incidence Rate Ratio; OR Odds Ratio; CI confidence interval; N number  *Attainment and unemployment were final (as opposed to yearly outcomes) therefore some children spent time in both care settings over the full study period | | | | | |  |  |  |  |  |
