# Supplementary figures and images for: Educational and health outcomes of schoolchildren in local authority care in Scotland: A retrospective record linkage study
Source: PLoS Med. 2021 Nov 12;18(11):e1003832. doi: 10.1371/journal.pmed.1003832 (PMC8589203; doi:10.1371/journal.pmed.1003832)

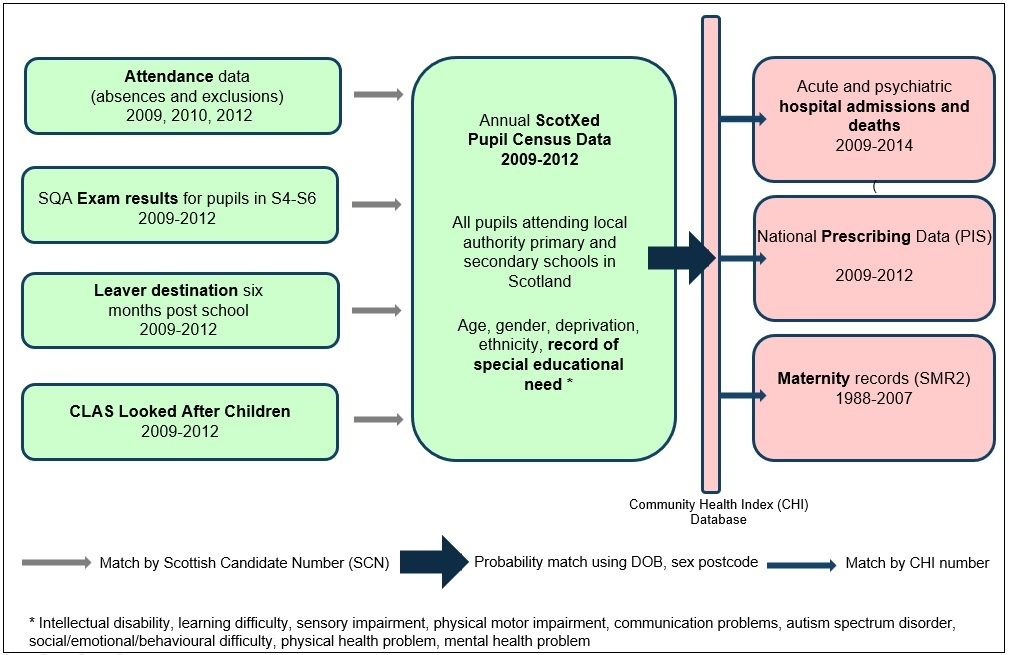

Supplement: S1 Fig — (TIF) [file pmed.1003832.s006.tif]

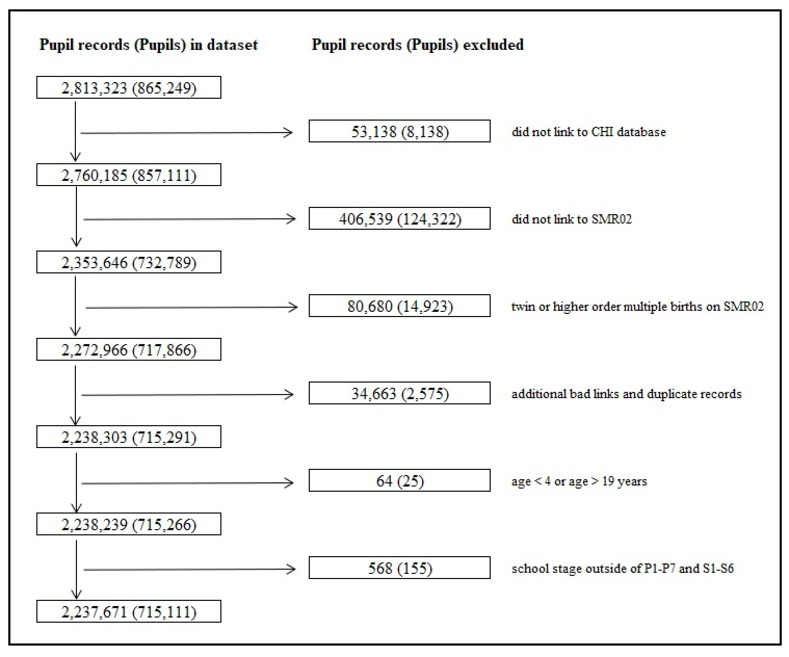

Supplement: S2 Fig — (TIF) [file pmed.1003832.s007.tif]
